# Estimation of Hypertension Risk from Lifestyle Factors and Health Profile: A Case Study

**DOI:** 10.1155/2014/761486

**Published:** 2014-06-15

**Authors:** Zhuoyuan Zheng, Ye Li, Yunpeng Cai

**Affiliations:** ^1^Shenzhen Institutes of Advanced Technology and Key Laboratory for Health Informatics, Chinese Academy of Sciences, Shenzhen University Town, 1068 Xueyuan Avenue, Shenzhen 518055, China; ^2^School of Computer Science and Engineering, Guilin University of Electronic Technology, Guilin 541004, China; ^3^University of Chinese Academy of Sciences, No. 19A Yuquan Road, Beijing 100049, China

## Abstract

Hypertension is a highly prevalent risk factor for cardiovascular disease and it can also lead to other diseases which seriously harm the human health. Screening the risks and finding a clinical model for estimating the risk of onset, maintenance, or the prognosis of hypertension are of great importance to the prevention or treatment of the disease, especially if the indicator can be derived from simple health profile. In this study, we investigate a chronic disease questionnaire data set of 6563 rural citizens in East China and find out a clinical signature that can assess the risk of hypertension easily and accurately. The signature achieves an accuracy of about 83% on the external test dataset, with an AUC of 0.91. Our study demonstrates that a combination of simple lifestyle features can sufficiently reflect the risk of hypertension onset. This finding provides potential guidance for disease prevention and control as well as development of home care and home-care technologies.

## 1. Introduction

Hypertension, also called high blood pressure (HBP), is a chronic medical condition which affects nearly 1 billion people worldwide and is directly accountable for the death of 9.4 million people per year by causing severe diseases including coronary heart disease, heart attack, heart failure, stroke, and kidney failure [1]. According to WHL and WHO, the best strategy to tackle this “silent killer” is to prevent it before it comes into being. Lifestyle factors, such as diet, smoking, or alcohol use, are known to have important impacts on hypertension onset. In recent years, the advent of mobile care technology made it possible to acquire the physiological features and monitor the life habits of a person easily in daily environments [2–4], which paves the way for personalized control and prevention of hypertension in home-care situations and surges great demands for creating models for hypertension risk estimation and management based on personal lifestyle factors and simple health profiles.

Although medical scientists have discovered scores of risk factors associated with the development of hypertension, investigations on systematic risk estimation or prediction are still lacking. One recent successful attempt of this type is the Framingham cohort study [5], which results in a risk prediction score for 1-, 2-, or 4-year hypertension onset composed of blood-pressure history, body mass index, parental history, and smoking habit. Despite its efficiency, the Framingham risk score barely comprises controllable lifestyle factors and thus provides little guidance for hypertension management. On the other hand, some recent researches also focus on assessing the impact of lifestyle and health profile factor on hypertension [6–10]. The randomized controlled trial of Whelton et al. [8] is aimed at determining the impact of weight loss or reduced sodium intake to hypertension treatment. The result showed that if sodium intake is reduced and weight is lost, there will be a feasible, effective, and safe nonpharmacologic therapy of hypertension for the old. Forman et al. [9] focused their research on women. They accessed hypertension incidences associated with dietary and lifestyle factors. And the result showed that adopting low-risk dietary and lifestyle factors has the potential of preventing a large proportion of new-onset hypertension occurring among young women. Wu et al. [10] carried out a cross sectional investigation on the prevalence of hypertension subtypes in adults of Tongshan County in Jiangsu Province, China, and studied the relationships between hypertension subtypes and related risk factors, indicating that the related risk factors have different levels of impact on the different hypertension subtypes. Nevertheless, existing works mainly explore associated factors without providing a complete picture about how lifestyle plays a role in hypertension. Although lifestyle modification pieces of advice are frequently given in hypertension treatments, few studies have ever been focusing on tracking to what extent these pieces of advice are followed, not to mention to what extent lifestyle affects the control or prevention of the disease [11].

In our study, we aim at creating a combined model that reflects the disease status of an individual patient based on lifestyle factors and simple health profile. With a large number of lifestyle factors investigated, not only can our model provide individual risk estimation suitable for health monitoring, but it also indicates the current most prevalent risk factors for a given person, thus shedding some light on active prevention of the disease via lifestyle management. Compared with traditional risk association studies, our model is able to capture interactions between multiple factors and extract a minimal set of independent factors that achieve high estimation accuracy. We find out a clinical model that can quantitatively assert the risk of hypertension from simple lifestyle features or health information that can be easily acquired without the intervention of complex medical instruments. This finding will provide advantages for the precaution or control of hypertension with the aid of home or mobile healthcare technologies. The model is obtained from the investigation of a chronic disease questionnaire data set for peasants of Pizhou City in Jiangsu Province and is generated via feature selection and logistic regression. A primary statistical analysis for the data set is performed in [12]. However, in this paper we go further to create a lifestyle related risk model using advanced methods. We evaluate the performance of the obtained model using external test data. Following the test protocol, the proposed model achieves an accuracy of 83.65% with an area under ROC curve (AUC) of 0.91, which justifies the possibility of monitoring hypertension risks from simple lifestyle profiles.

The materials of the paper are organized as follows.  Section 2 introduces materials and describes the experiment designs for data analysis in the paper.  Section 3 gives our experiment results. In Section 4, we present our discussion on the last section. Finally, we conclude this paper.

## 2. Methods

### 2.1. Materials

#### 2.1.1. Data Collection

The questionnaire is designed for risk factors investigation of chronic disease of rural areas in Pizhou City. The Health Bureau of Pizhou City was in charge of this survey; it organized interviewers and coordinated the whole process. The survey was conducted during October 2008 to January 2009. 6563 participants (3230 males and 3333 females) from 18 administrative villages in 5 towns of Pizhou City were interviewed. The data collection procedures were in accordance with the Declaration of Helsinki and were approved by the ethics committee of Shenzhen Institutes of Advanced Technology. Each participant signed and gave informed consent of data collection prior to data acquisition.

All 112 interviewers, including rural doctors of country clinics in the participants' residential area and nurses or physicians in local hospitals, were trained to take measurements and record the result. At the same time they were asked to guide and help the participants to finish the questionnaire correctly. In order to make sure of the consistency of the measurement among different participants, the same standards, including the accuracy of measurement and the types of instruments, were adopted. Each interview was carried out by a team consisting of two interviewers: one is responsible for measuring blood pressure (BP), arterial pulse, and fasting plasma glucose (FPG) level and the other is responsible for assisting interviewee to fill out questionnaires when needed. 6340 questionnaires were conducted during home visit, which account for 96.6% of all. The remaining 223 were finished in public places including clinic or hospital. After the participant had been resting in a seated position for 5 minutes, three measurements of systolic blood pressure (SBP) and diastolic blood pressure (DBP) were taken with at least 2-minute intervals on the arm with digital blood pressure monitor, which can give out SBP, DBP, and arterial pulse, simultaneously. Blood glucose tester was employed to measure participants' FPG in 12 hours after dinner.

In the questionnaire, information about chronic diseases (high blood pressure, coronary heart disease, stroke, diabetes, and malignant tumors) was enquired and included a total of 11 sections (personal information, living condition, health care, chronic history, familial chronic history, smoking, drink, alcohol use, eating habits, daily living and physical exercise, woman menstruation, and birth history) comprising altogether 221 factors. Factors associated with personal identity are excluded from the study. Also, to prevent information leaks introduced by concurrent symptoms, we excluded other chronic diseases and personal and family medical history from the study. Finally, we filtered out factors with over 90% of missing values. After filtering, 76 factors are kept as variables in the consequent study.

#### 2.1.2. Data Extraction

The data collected include three measurements on SBP and DBP, respectively. In order to assure the accuracy of the labeling, we extract only patient data with clear and consistent clinical outcomes. According to the guideline standard [13], HBP patients are identified on the basis that three measured values of SBP are higher than 140 mmHg and three measured values of DBP are higher than 90 mmHg, or those taking antihypertensive drugs. For samples with age younger than 18, we refer to standards developed by Mi et al. [14]. At the same time, nonhypertension data was extracted on the basis that three measured values of SBP are less than 120 mmHg and three measured values of DBP are less than 80 mmHg. In this way, 1785 records, including 352 hypertension and 1433 nonhypertension records, are extracted. We randomly sampled half of the records (176 hypertension and 716 healthy) to form a training dataset and use the rest as the test dataset.

The remaining 4778 samples are usually called borderline HBP in academy. In our study, with the exception of the selected 1785 records above, the borderline HBP samples were used to validate our final model. We divided these samples into four groups. First, we picked out samples exhibiting normal blood pressure at least one measurement as so-called white-coat HBP. And 2668 samples belong to this group. Next, the remaining 2110 samples were divided into three groups according to the severity of suffering from HBP. We named three groups HBP1, HBP2, and HBP3, respectively. If for all three measurements of one sample in each measurement its SBP is higher than 140 mmHg or DBP is higher than 90 mmHg, this sample should be put into HBP3. There are 324 samples in HBP3. Then, we classify all samples whose SBP is higher than 140 mmHg and DBP is higher than 90 mmHg one or two times into HBP2. And 127 samples fall into this group. Finally, the remaining 1659 samples are included in group HBP1.

### 2.2. Experiment Designs and Protocols

**Figure 1:**
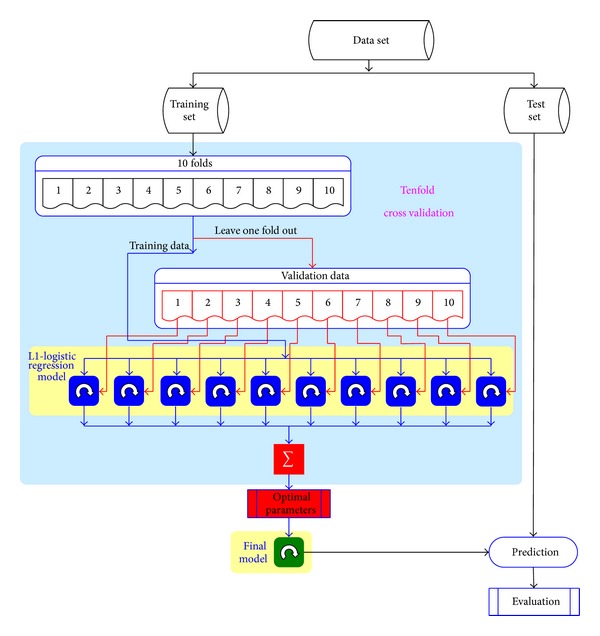
Data analysis flow for our experiments. Data set is evenly divided into training and test set.

The data analysis procedure is depicted in Figure 1. The whole scheme begins with data extraction and ends with the result of ROC curve, AUC for evaluation, and a list of clinical signatures. The entire procedure is implemented in Matlab.

Because the measurement of fasting plasma glucose (FPG) is invasive, which is not desired in our purpose, we carry out the data analysis procedure twice, one with FPG included and one without, and compare the performance of the derived model in both situations.

#### 2.2.1. Preprocessing

Real world data are generally incomplete and inconsistent dirty data. Data preprocessing can improve data quality and reduce processing time.

(*1) Normalization. *Normalization may improve the efficiency of data analysis algorithms and balance the effect of each factor in case of linear analysis. In our study, we apply the *z*-score normalization for all fields. This scheme can be expected to perform well if prior knowledge about the average score and the score variations of the matcher is available [15].

In *z*-score normalization, the values for an attribute *A* are normalized based on the mean and standard deviation of *A*. A value *v* of *A* is normalized to *v*′ by computing

(1) $$\begin{array}{c} v^{\prime }=\frac{v-\overset{-}{A}}{\sigma_{A}}, \end{array}$$

where $\overset{-}{A}$ and *σ*_*A*_ are the arithmetic mean and the standard deviation, respectively, of attribute *A*.

(*2) Missing Value Imputation. *Questionnaire data usually comprises missing values in some fields due to various artificial malfunctions, and these missing values fit the assumption of “missing at random” in statistical concept. We use two different policies of missing value imputation, one filling with average value and one with default value, depending on the characteristics of each field and the design of the questionnaire. For example, we take the average value of height as imputed value, but for drinking white liquor, we use the default value 0. The reason is that according to the culture context not filling the field “years of drinking” usually means that the investigated person is not a liquor-drinker rather than that he is not willing to say it.

#### 2.2.2. L1-Regularized Logistic Regression with Sample Balancing

Logistic regression is a multivariable method that was devised for dichotomous outcomes. It is particularly appropriate for models involving disease state (diseased/healthy) and decision making (yes/no) and therefore is widely used in studies in the health sciences [16].

In logistic regression model, the probability of an outcome is related to a series of potential predictor variables by an equation of the form *b* and **w** = (*w*_1_, *w*_2_,…, *w*_*i*_). Consider

(2) $$\begin{array}{c} \ln\left(\frac{p}{1-p}\right)=b+w_{1}x_{1}+w_{2}x_{2}+\cdots +w_{i}x_{i}\; \end{array}$$

or

(3) $$\begin{array}{c} p=\frac{e^{b+w_{1}x_{1}+w_{2}x_{2}+\cdots +w_{i}x_{i}}}{e^{b+w_{1}x_{1}+w_{2}x_{2}+\cdots +w_{i}x_{i}}+1}, \end{array}$$

where *p* is the probability of the outcome of interest, *b* is an intercept term, (*w*_1_, *w*_2_,…, *w*_*i*_) are thecoefficients **w** associated with each variable, (*x*_1_, *x*_2_,…, *x*_*i*_) are the values of the potential predictor variables, and *i* is a unique subscript denoting each variable [17, 18]. Here *x*_1_, *x*_2_,…, *x*_*i*_ represent the factors in a patient profile. In the resulting model, the coefficients of the predictor variables are interpreted as signifying the relative contribution of their respective variables toward the predicted probability of a positive outcome [16].

Classical logistic regression model is known to be vulnerable to multiple numerical issues including collinearity or ill-conditioned data. Regularization is a standard technique to tackle these problems. On the other hand, for medical data, especially questionnaire data, most of the factors investigated are not necessarily connected with the target disease. The existence of many irrelevant factors increases the difficulty of solving the regression model and often leads to bad performance. Feature selection is required as a preprocess step in analyzing these types of data.

In our paper, we address both problems using the technique of L1-regularized logistic regression. Logistic regression with L1 regularization or so-called sparse logistic regression [19, 20], where the weight vector of the classifier has a small number of nonzero values, has been shown to have attractive properties such as feature selection and robustness to noise and proposed as a promising method for feature selection in classification problems. Several specialized solution methods have been proposed for L1-regularized logistic regression problems (LRPs) [21]. We used L1-regularized logistic regression as a tool of building discriminative models. Theoretically, it is proved that L1-regularized learning has a low sample complexity, which enables it to learn a correct model with relatively limited number of instances and minimizes the risk of false discovery. We also verified the obtained model using the permutation test, which confirmed that the risk of false discovery is very low and the derived estimation model is reliable.

Taking into account that the number of healthy samples in our investigation is a few times more than the HBP ones, we use the following formulation of L1-regularized logistic regression:

(4) min⁡w,b1∑αi∑i=1nαilog⁡(1+exp⁡(−yi(wTxi+b)))+λ||w||1,

(5) αi={m−m++m−,when  i  is  a  negative  sample, m+m++m−,when  i  is  a  positive  sample.

Here *α*_*i*_ is a sample weight that balances the impact of healthy and patient samples and *m* ^+^ and *m* ^−^ are the number of positive and negative samples, respectively. *λ* is a regularization parameter tuned with cross validation described before in this section, **w** = (*w*_1_, *w*_2_,…, *w*_*i*_) is the prediction weight showing the prediction strength and whether a factor is a predisposing or suppression one (positive for predisposing factors and negative for suppression factors), and *b* is the decision threshold. Given a new patient $\overset{-}{x}$, the prediction score $w^{T}\overset{-}{x}$ indicates the risk of suffering from hypertension. A higher score represents a higher trend of suffering from hypertension. The threshold *b* gives approximately the borderline between the scores of normal and hypertension profiles. Equation (4) can be solved using the method described in [22].

#### 2.2.3. Tenfold Cross Validation

Model building algorithms usually involve the selection of a few parameters. In L1-regularized logistic regression, the regression parameter *λ* needs to be predetermined before training the final model. Cross validation is a standard procedure in data mining and machine learning fields that determines the optimal value of the parameters without human intervention. By ruling out the bias of artificial selection, the reproducibility and generality of the results can be assured. Cross validation allows estimation of the prediction error of a model by cyclically leaving out a portion of the data as an independent validation set [23] and the rest as the training set. In each validation step, a model is trained with the training set alone and the performance is tested on the validation set. After the entire data set is tested, the overall performance on all cycles can be regarded as a fair estimation about the quality of the achieved model when applying to novel data sets.

Kohavi's results indicate that tenfold cross validation is the best method for model selection, even if the computation power allows using more fold [24]. With tenfold cross validation, the data are divided into 10 equal parts; the model is developed on 9/10 of the data (i.e., the training sets) and then evaluated on the remaining 1/10 of the data (i.e., the independent test set). This is repeated for each possible 9/10 and 1/10 of the data and the resulting ten prediction errors are averaged.

In this paper, the studied dataset is highly imbalanced for healthy versus HBP samples, which will lead to disturbing in random sampling and degrade the performance of cross validation. To overcome this effect, we fix the proportion of hypertension to nonhypertension cases during sampling. We divide hypertension and nonhypertension case into 10 equal parts, respectively. Then one part is picked out from hypertension and nonhypertension case, respectively. These two parts are merged into one part. By this way, we can get 10 parts with the same proportion of hypertension to nonhypertension case.

The receiver operating characteristics (ROC) curve [25] is used to measure the performance of the created model in both the model selection and the external test. A ROC curve is a plot of sensitivity (true positive rate, TPR) on the *y*-axis against 1-specificity (false positive rate, FPR) on the *x*-axis for varying values of the threshold (cutoff) value. Sensitivity and specificity [26] are the basic measures of the accuracy of a diagnostic test, which describe the abilities of a test to enable one to correctly diagnose disease when disease is actually present and to correctly rule out disease when it is truly absent. The accuracy of a test is measured by comparing the results of the test to the true disease status of the patient. The 45° diagonal line connecting (0, 0) to (1, 1) is the ROC curve corresponding to random chance. The ROC curve for the gold standard [27] is the line connecting (0, 0) to (0, 1) and (0, 1) to (1, 1). The area under curve (AUC) [28–30] is a summary measure that essentially averages diagnostic accuracy across the spectrum of test values, which is defined as

(6) $$\begin{array}{c} \text{AUC}=\overset{n}{\underset{i=2}{\sum }}\text{AU}{\text{C}}_{i}=\overset{n}{\underset{i=2}{\sum }}\left(\text{FP}{\text{R}}_{i}-\text{FP}{\text{R}}_{i-1}\right)\times \text{TP}{\text{R}}_{i}. \end{array}$$

An ideal prediction model would have an AUC of 1, whereas a random guess would have an AUC of 0.5. The AUC criterion has been widely adopted in CVD risk prediction [31, 32].

#### 2.2.4. Permutation Test

Permutation test is a standard statistical method that asserts the risk of false discovery of an analysis method. In the case of small data set, it can be frequently observed that a prediction model performs accurately on the training data set but poorly on novel data, because the employed analysis method is too powerful in fitting the training data and overexplores some random confounding factors that are of no interest to investigators. Permutation tests are especially useful and relevant for multivariate analysis, where distributional assumptions are even more difficult to fulfill [33].

The idea of permutation test is to keep all experiment protocols and analysis methods fixed except that the sample labels are all randomly permutated and to compare the results obtained by the asserted analysis method using real and permutated labels. The *p*-value, also called the false discovery rate (FDR), is defined as the probability that the prediction quality (AUC value in this case) of a random test is equal to or greater than the one with true labels. A lower *p*-value suggests that the prediction model obtained using real labels has more probability to be a correct model, rather than conduct to false discoveries.

## 3. Results

In our model, field HBP acts as target variable and the rest are predictor variables. According to formula (2), we can calculate the probability *p*, indicating the probability that people (case) suffer from hypertension, for every case in test sets. If for one case *p* is greater than 0.5, this shows that this case has higher chance developing hypertension and we have reason to believe that this is a hypertension case. By comparing the HBP fields of every case in test sets with their corresponding *p* value calculated, we validate the fitness of our model.

Note that the estimation score, $\text{pred}=w^{T}\overset{-}{x}+b$, is equal to the value ln⁡[*p*/(1 − *p*)] and grows monotonously with *p*, with an attracting character that pred = 0 when *p* = 0.5. Hence, we can use pred as a simple indicator for hypertension. Given a patient profile *x*_1_, *x*_2_,…, *x*_*i*_, we calculate the estimation score $\text{pred}=w^{T}\overset{-}{x}+b$ to estimate the hypertension status of the given patient and measure the performance of the obtained model on the test set. We used both the AUC criteria and the confusion matrix to measure the performance. In computing the estimation accuracy, we used a threshold pred = 0 to determine the clinical outcome of the models. Samples with pred > 0 are assigned hypertension estimations, and others are assigned nonhypertension estimations, which are then compared with the true status. To plot the ROC curve, we sort the estimation scores on all instances from largest to smallest, forming a series of threshold values. At each instance value a measuring pair (*1-specificity, sensitivity*) is calculated and plotted on the curve.

**Figure 2:**
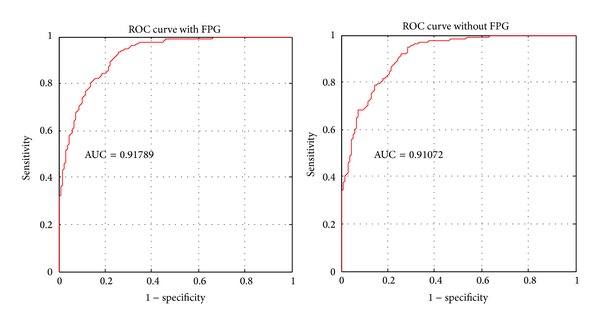
ROC curve of validation result on test set with and without FPG. AUC with FPG is 0.91789 and its standard error is 0.01481. AUC without FPG is 0.91072 and its standard error is 0.01537.

**Figure 3:**
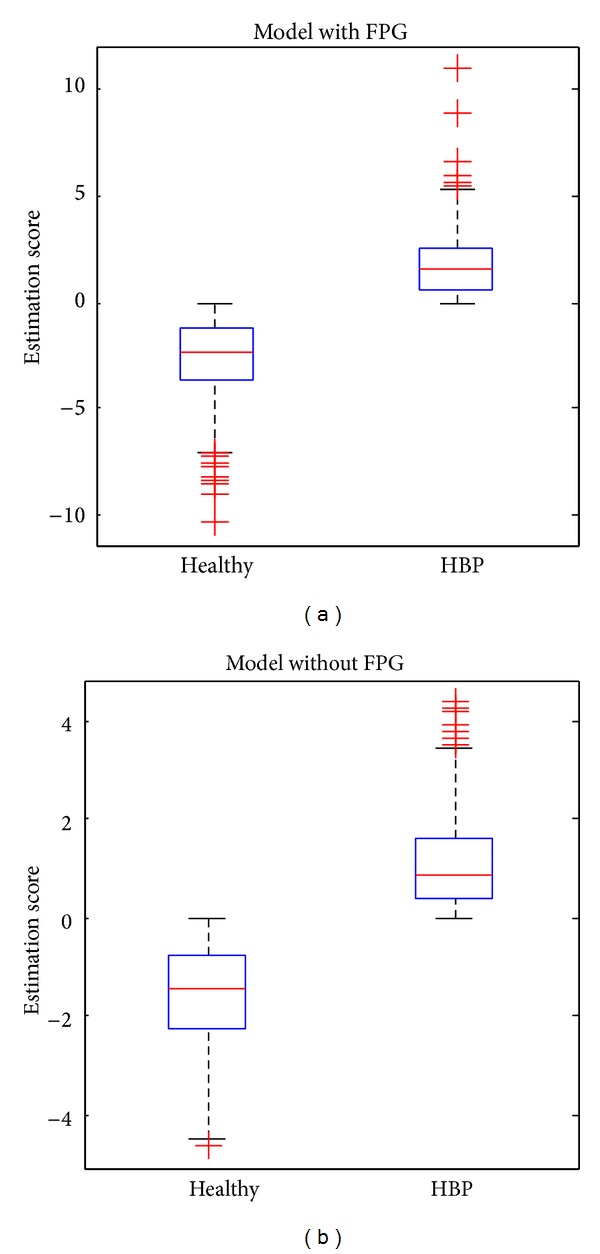
Box plots of the distribution of estimation scores for the healthy and HBP groups. (a) Model with FPG and (b) model without FPG.

We build two different models with and without FPG, respectively. The evaluated ROC curves on the independent test set for the two models are depicted in Figure 2. The AUC is 0.91789 for the model with FPG and 0.91072 without FPG. Their corresponding standard errors (SE) are 0.014805 and 0.015371, respectively. We see that both models accurately infer the hypertension status of the patients from their health profiles. The box plots of the estimation scores on the test samples in both cases are given in Figure 3.  Table 1 presents the confusion matrices in values of sensitivity and specificity for both models, respectively. For the data with FPG, the overall test accuracy of the model is 83.65% while for the one without FPG the accuracy is 82.87%.

**Figure 4:**
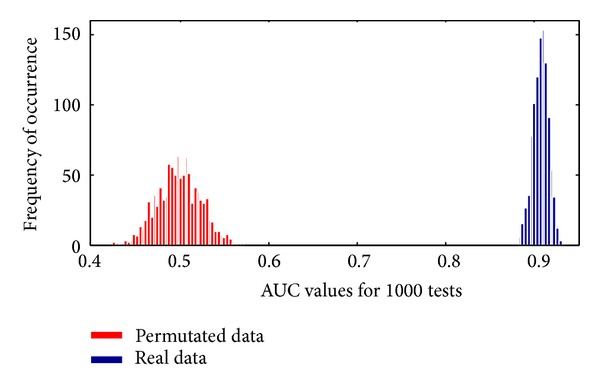
Results of permutation tests and randomization tests. We see that the AUC values achieved on real data clearly outperform those on permutated data, which suggests that the models have a good performance. The deviation of the AUC values on real data is small, which suggests that the performance of the proposed method is quite stable against random variations.

To perform permutation test, we generated 1000 independently permutated datasets and carried out tests on them. Additionally, we also repeat the tests on real data by randomly repartitioning the training and test sets. For consideration of the invasiveness of FPG, we adopt the model without FPG in this test. The comparison of the AUC scores between permutated and real data is shown in Figure 4. It turned out that the performances on the randomly labeled data sets were obviously inferior to those on the true data set. We can claim that, at the nominal level *α* = 0.01, the obtained signature is statistically significant and is not a false discovery. On randomly labeled data, the average AUC for 1000 permutation tests is 0.499 (which is 0.5 in ideal case) and the best score is 0.57, while, on real data, the average AUC for 1000 estimation tests is 0.908, with the minimal score of 0.88. This confirms that the model building protocol suggested in this paper is not biased or overfit to the data and the obtained results are highly reliable.

Although FPG is a very important and useful indicator of hypertension, the above result shows that an alternative indicator can be constructed with no adoption of FPG measurement and without significant loss of accuracy. This finding suggests that a surrogate model can be used to assess the risk of hypertension without collecting all important factors that affect the disease, which is quite helpful for our idea of constructing a clinical signature from very simple health profiles that are suitable for home-care or mobile-care technologies.

In order to study the contribution of each factor to the clinical model and reduce accidental error, we repeat the experiment protocol on 100 randomly partitioned training/test sets and calculate all coefficients (*w*_1_, *w*_2_,…, *w*_*i*_) associated with each field. These coefficients **w** are extracted from the original results. In order to make a fair comparison of different factors in each model, for each run, we normalize the coefficients through dividing them to the sum of absolute value of all coefficients. Then, we calculate the mean value of the coefficients for each factor in 100 runs and use it as the estimation strength of the factor. We only keep factors with absolute average weights greater than 0.01. The average normalized coefficients of the selected factors and their distribution ranges are given in Table 2. Factors that show a positive correlation with the onset of hypertension are marked in bold and negative ones are marked in italic. With L1 regularization, only a near-minimal set of factors is selected among lots of informative variables (*p* < 0.01), which reduces the risk of false discoveries. On the other hand, L1 logistic regression explores the interaction between variables and includes some useful factors which seem to be noninformative when assessed individually (*p* > 0.05). These can be hardly achieved using traditional forward or backward selection methods.

**Figure 5:**
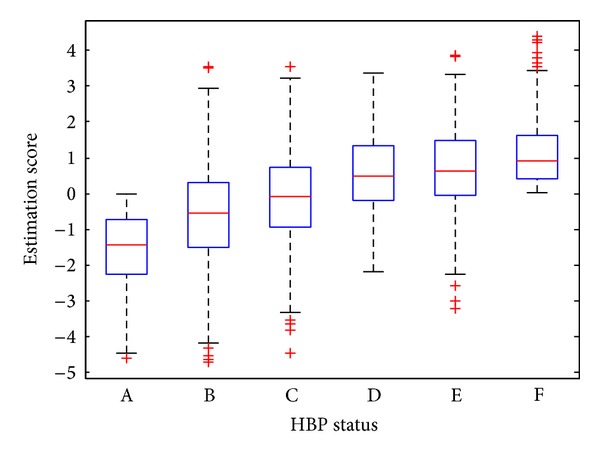
Box plots of the distribution of estimation scores for samples of different HBP status. The model without FPG is used. (A) Healthy, (B) suspected white-coat HBP, (C) borderline 1, (D) borderline 2, (E) quasi-HBP, and (F) HBP.

In our experiments, borderline HBP samples are used to verify the effectiveness of our model. According to our grouping method, we can sort these groups by the risk of suffering from HBP. The risk order from low to high is white-coat HBP, HBP1, HBP2, and HBP3. We calculate the estimation score of different groups, including healthy and HBP samples in test dataset, through our final model. For the same consideration of permutation test, we also adopt the model without FPG. Box plot for all six group samples is shown in Figure 5. From the distribution of the estimation score, we can conclude that our model can accurately predict the risk of hypertension for different populations. The healthy is of the lowest risk and HBP is of the highest.

As is consistent with previous researches [7–10], age, arterial pulse, FPG, body mass index, sleep quality, intake of salt, oil, liquor, and pickles are the prevailing factors that affect the onset of hypertension. In the absence of FPG information, several diet-related factors (e.g., amount, cost of food intake, and type of meat intake) take up the role and yield almost equally good prediction; this illustrates how a surrogate signature can work in risk screening without deep knowledge about the mechanism of the disease. The model indicates that intake of animal oil in a small amount helps reduce the risk of hypertension. This should be interpreted under the background that the vast majority of oil consumption for the investigated group of people is formed up by plant oils. From the model we also see that divorced people are more likely to develop hypertension compared with single or married people; and women bearing children many times are also associated with higher risks. Both phenomena can attribute to the tensions in daily life.

In the model built without FPG information, lifestyle factors possess 32% of the prediction weights, which is quite a significant composition even if we neglect the indirect impact of lifestyles through health profiles. Since the extracted factors in the model are mostly long-term stable and directly or indirectly controllable, it is promising that they do comprise relevant features that can be used as preceding warning signals for active prevention of hypertension. Our future study will focus on exploring more accurate, predictive, and administrable factors and reach a lifestyle evaluation metric that monitors and alerts healthy people from risks of hypertension.

## 4. Discussion

Hypertension is known to be a hybridized disease caused by various genetic mutations, congenital defects, or acquired metabolic or cardiovascular disorders. Despite these heterogeneities, in this study we show that a surrogate signature does exist to reflect the risk of hypertension for a normal individual, and the signature can be formed up by quite simple health profile and lifestyle features. Unlike previous association study approaches, our method directly presents a risk estimation result to each individual patient. This is very beneficial in providing a guidance indicator for disease prevention and surveillance.

It should be emphasized that, as a surrogate signature obtained by cross sectional study, the features used in the model are not necessarily the direct cause of hypertension. Nevertheless, most of the features extracted in this paper are directly or indirectly controllable, which serve as ideal targets for early intervention of cardiovascular diseases. Our study also shows that living style plays an active role in protecting cardiovascular health. This replicates the common idea that modification of lifestyle helps to prevent hypertension. More clinical experiments and investigations, especially longitudinal studies with follow-up responses, should be carried out to further verify the underlying mechanism about how the change of lifestyle can influence the risk prognosis.

Lifestyle pattern is a combination of complex behaviour associated with multiple historical, geological, economic, cultural, and ethical influences. It is still unclear whether the clinical model proposed in this study reflects the pervasive effects of lifestyle to hypertension or merely depicts the local regularity in a group of people sharing common major lifestyles but with some variations. Comparative studies should be carried out to testify the replicability of the derived model in people with different geological or ethical backgrounds. With more data collected, the clinical model can be better optimized and become more pervasive. This will be one of our future directions.

## 5. Conclusions

Hypertension is considered a major threat to human health by causing severe circulation or metabolic diseases. Lifestyle modification has long been considered a promising manner for disease control and prevention. A comprehensive model about how lifestyle factors are associated with the total risk of hypertension is a mandatory demand for effective health management, which is not yet addressed by existing studies. In this paper, we demonstrated that a combination of controllable lifestyle factors and simple health profile can accurately reflect the hypertension status of a person. This enables a manner of controlling and preventing the onset of the disease by actively monitoring lifestyle factors using mobile-care technologies, assessing the overall risk, and identifying the prevailing hazard factors, which will be a useful step towards efficient hypertension prevention.

Home-care and mobile-care devices are becoming more and more popular in social life, providing more convenient and real-time monitoring of individual health. Meanwhile, due to the restriction of cost and portability, these devices are not expected to be either complex or expensive, which restricts their abilities of disease diagnosis. Our investigation suggests that the organization of simple, noninvasive health monitoring can lead to accurate precaution of important diseases, which provides support for wider adoption of home-care or mobile-care in promoting public health. Specifically, home-care electronic devices can be equipped to collect basic physiological parameters of individuals and monitor the diet patterns, exercise frequencies, and other habits of a family periodically, with low cost and in a noninvasive manner. A risk score can be then evaluated using the derived model and an alert can be delivered if an increasing trend of risk towards hypertension is observed for an individual. By this means, active prevention and monitoring of HBP by lifestyle modification can be achieved.

## Figures and Tables

**Table:** (a) The overall accuracy is 83.65%

	Actual value	
	Positive	Negative
Prediction value		
Positive	146	116
Negative	30	601
Accuracy	82.95%	83.82%

**Table:** (b) The overall accuracy is 82.87%

	Actual value	
	Positive	Negative
Prediction value		
Positive	114	121
Negative	32	596
Accuracy	81.82%	83.12%

**Table 2:** Factors that affect HBP and their weights.

Factor name	Normalized weight		Original value					
	With FPG	Without FPG	Imputed values	Avg	Std	Max	Min	Units
**Arterial pulse**	**0.116**	**0.118**	**74.79**	**74.79**	**6.48**	**119**	**56**	**Beats/min**
**Age**	**0.115**	**0.108**	**38**	**38**	**15.62**	**68**	**14**	**Year**
**FPG**	**0.069**	***✗***	**4.73**	**4.73**	**0.9**	**15.2**	**2.1**	**mmol/L**
**Freq. eating pickles**	**0.062**	**0.061**	**1/m**	**[1/m or less, 2~3/m, 1~2/w, 3~6/w, >=7/w]** ∗				
**BMI**	**0.058**	**0.058**	**22.56**	**22.56**	**2.83**	**39.05**	**14.7**	**kg/m** ^ 2^
*Height *	*−0.057 *	*−0.061 *	*163.21 *	*163.21 *	*7.83 *	*184 *	*120 *	*cm *
**Waistline**	**0.057**	**0.057**	**77.99**	**77.99**	**8.89**	**111**	**51**	**cm**
**Hip circum.**	**0.057**	**0.045**	**88.75**	**88.75**	**10.56**	**137**	**62**	**cm**
**Salt intake**	**0.028**	**0.026**	**0.56**	**0.56**	**0.28**	**3.5**	**0.15**	**500 g/m**
**Years of drinking**	**0.028**	**0.025**	**0**	**2.56**	**7.72**	**51**	**0**	**Year**
*Intake of animal oil *	*−0.026 *	*−0.033 *	*0.14 *	*0.14 *	*0.39 *	*4 *	*0 *	*500 g/m *
*Sleep quality *	*−0.023 *	*−0.023 *	*Ordinary *	*[bad, ordinary, good] *				
*Female menstruation regularity *	*−0.02 *	*−0.023 *	*1 *	*— *	*— *	*— *	*— *	*Boolean *
**Divorced**	**0.019**	**0.022**	**0**	**—**	**—**	**—**	**—**	**Boolean**
**Intake of plant oil**	**0.018**	**0.022**	**3.09**	**3.09**	**1.28**	**15**	**0.15**	**500 g/m**
*Work-time physical activity *	*−0.016 *	*−0.025 *	*Median *	*[rare, light, median, heavy, extremely heavy] *				
**Drinking white liquor**	**0.015**	**0.015**	**0**	**0.97**	**2.56**	**43**	**0**	**Times/w**
**Drinking tea**	**0.015**	**0.014**	**Occasional**	**[none, occasional, often]**				
**Times of bearing**	**0.015**	**0.016**	**0**	**1**	**1.38**	**10**	**0**	**Times**
**Family food expenditure per month**	**0.014**	**0.024**	**861.04**	**861.04**	**694.95**	**6000**	**50**	**RMB**
**Meat intake type**	**0**	**0.01**	**no meat**	**[no meat, lean, half fat and half lean, fat]**				
**Staple food intake**	**0**	**0.013**	**13.67**	**13.67**	**4.39**	**30**	**4**	**50 g/d**
**Times of abortion**	**0**	**0.011**	**0**	**0.04**	**0.28**	**5**	**0**	**Times**

Weights are the mean value of 100 prediction tests.

For quantization values, for example, sleep quality, its corresponding original values are enumerated in original value column.

∗/m means ~per month and /w, /d means ~per week, ~per day, respectively.

Unit Boolean means that this attribute value is of Boolean type, value 1 refers to true and value 0 refers to false.
